# Accelerated brain age in Moyamoya disease patients: a deep learning approach and correlation with disease severity

**DOI:** 10.3389/fnins.2025.1668993

**Published:** 2025-09-25

**Authors:** Wenjie Li, Suhua Chen, Xin Chen, Xiangtian Ji, Huan Zhu, Qihang Zhang, Chenyu Zhu, Tao Wang, Yan Zhang, Jun Yang

**Affiliations:** ^1^Department of Neurosurgery, Peking University Third Hospital, Beijing, China; ^2^Department of Neurosurgery, Beijing Tiantan Hospital, Capital Medical University, Beijing, China; ^3^Center for Precision Neurosurgery and Oncology, Peking University Health Science Center, Beijing, China

**Keywords:** Moyamoya disease, brain age, DenseNet architecture, deep learning, magnetic resonance imaging

## Abstract

**Introduction:**

This study aims to utilize a DenseNet based deep learning framework to predict brain age in patients with Moyamoya disease (MMD), examining the relationship between brain age and disease severity to enhance diagnostic and prognostic capabilities.

**Methods:**

We analyzed unenhanced MRI scans from 432 adult MMD patients and 565 normal controls collected between January 2018 and December 2022. Data preprocessing involved converting DICOM files to NIFTI format and labeling based on established diagnostic criteria. A DenseNet121 architecture, implemented using PyTorch, was employed to predict brain age. Statistical analyses included correlation assessments and comparisons between predicted brain age, chronological age, and MRA scores.

**Results:**

The predicted brain age for MMD patients was significantly higher than their chronological age, averaging 37.9 years versus 35.8 years (*p* < 0.01). For normal controls, predicted brain age matched chronological age at 36.5 years. Delta age (difference between predicted brain age and chronological age) was significantly elevated in MMD patients (*p* < 0.001) and positively correlated with MRA scores, indicating a link between arterial stenosis severity and accelerated brain aging.

**Discussion:**

The DenseNet based model effectively predicts brain age, revealing that MMD patients experience accelerated brain aging correlated with disease severity. These findings highlight the potential of brain age prediction as a biomarker for MMD, aiding in personalized treatment strategies and early intervention. Future research should explore multi-center datasets and longitudinal data to validate and extend these findings.

## Introduction

Moyamoya disease is a rare cerebrovascular disease characterized by gradual narrowing of the internal carotid artery and its main branches (Scott and Smith, 2009), resulting in a decrease in blood flow to specific areas of the brain (Tagawa et al., 1987). This type of cerebral hypoperfusion can lead to reduced oxygen and nutrient transport, neuronal loss, and progressive brain tissue damage (Taki et al., 1988; Kazumata et al., 2015; Hara et al., 2018; Baron et al., 2014). Therefore, over time, structural and cognitive changes occur, exacerbating as the disease progresses (Su et al., 2013; Kazumata et al., 2019; Qiao et al., 2017). Cognitive function, to a certain extent, reflects the severity of the disease. The testing of cognitive function relies on the Mini Mental State Examination (MMSE), which has a high sensitivity to mild cognitive impairment (Tsunoda et al., 2023). However, the false-positive rate of MMSE is high, making it difficult to effectively guide clinical treatment (Mitchell, 2013). Cerebral revascularization plays a certain role in the treatment of cognitive impairment in moyamoya disease (Bao et al., 2022; Kim et al., 2016). In the field of neuroscience, the importance of brain structure magnetic resonance imaging has been recognized as it can reveal atrophy in different regions of the brain (Giedd, 2004). The gray matter volume of the brain in patients with moyamoya disease decreases to varying degrees and is closely related to cognitive function (Ramanoël et al., 2018). Based on the characteristics of research methods, research is limited and cannot be evaluated for individuals. The concept of “brain age” has emerged and is receiving increasing attention. It is a means of estimating the age of an individual's brain based on a series of structural neuroimaging features (Smith et al., 2019). This concept is used to evaluate the degree of aging of the brain relative to a person's actual age. The deviation between an individual's brain predicted age and their physiological age—also known as brain predicted age difference (brain-PAD) or delta age can be used to quantify the deviation from healthy aging (Cole and Franke, 2017). Although the normal aging process affects the brain to some extent, pathological factors can accelerate this aging process (Yankner et al., 2008). The latest advances in machine learning have enabled the creation of brain age prediction models that utilize complex, nonlinear, and multivariate imaging data (Baecker et al., 2021). However, existing models typically require high-resolution T1 images and impose strict requirements on scanner providers and imaging protocols, which rely on extensive data preprocessing (Jonsson et al., 2019; Franke et al., 2010). The development of deep learning technology has opened up avenues for processing large, high-dimensional, and nonlinear datasets, expanding the scope of brain age prediction. DenseNet is a type of convolutional neural network (CNN) architecture. DenseNet differs from traditional CNN architectures by promoting maximum information flow between layers. In a typical CNN architecture, each layer receives input only from the preceding layer. However, in DenseNet, each layer receives input from all preceding layers. This dense connectivity pattern ensures that features learned by the network at any depth are directly available to all subsequent layers. It encourages feature reuse, which helps combat the vanishing gradient problem and enables better gradient flow during training. It reduces the number of parameters compared to traditional CNN architectures, leading to more efficient models. Dense connectivity facilitates feature propagation and enhances feature extraction, which can lead to improved performance, especially in tasks with limited data. Due to these advantages, DenseNet has become a popular choice for various computer vision tasks, including image classification, object detection, and image segmentation (Iandola et al., 2014). A brain age framework suitable for routine clinical head MRI examinations has been constructed. Based on DenseNet, rapid and accurate assessment of brain age in patients with moyamoya disease can be achieved (Wood et al., 2022). Given the age-dependent clinical manifestations and progression of moyamoya disease, accurate prediction of brain age is crucial. Accurate brain age estimation helps clinicians classify patients based on the severity of age-related symptoms and helps develop appropriate treatment strategies. Early detection through brain age prediction may promote timely intervention and potentially prevent serious complications, including stroke. In clinical practice, age prediction helps to customize diagnosis and treatment methods, ensuring personalized and effective patient care. Therefore, this study adopts a deep learning brain age prediction framework to predict brain age in patients with Moyamoya disease and elucidates the complex relationship between brain age and disease severity.

## Methods

### Data collection

**Figure 1:**
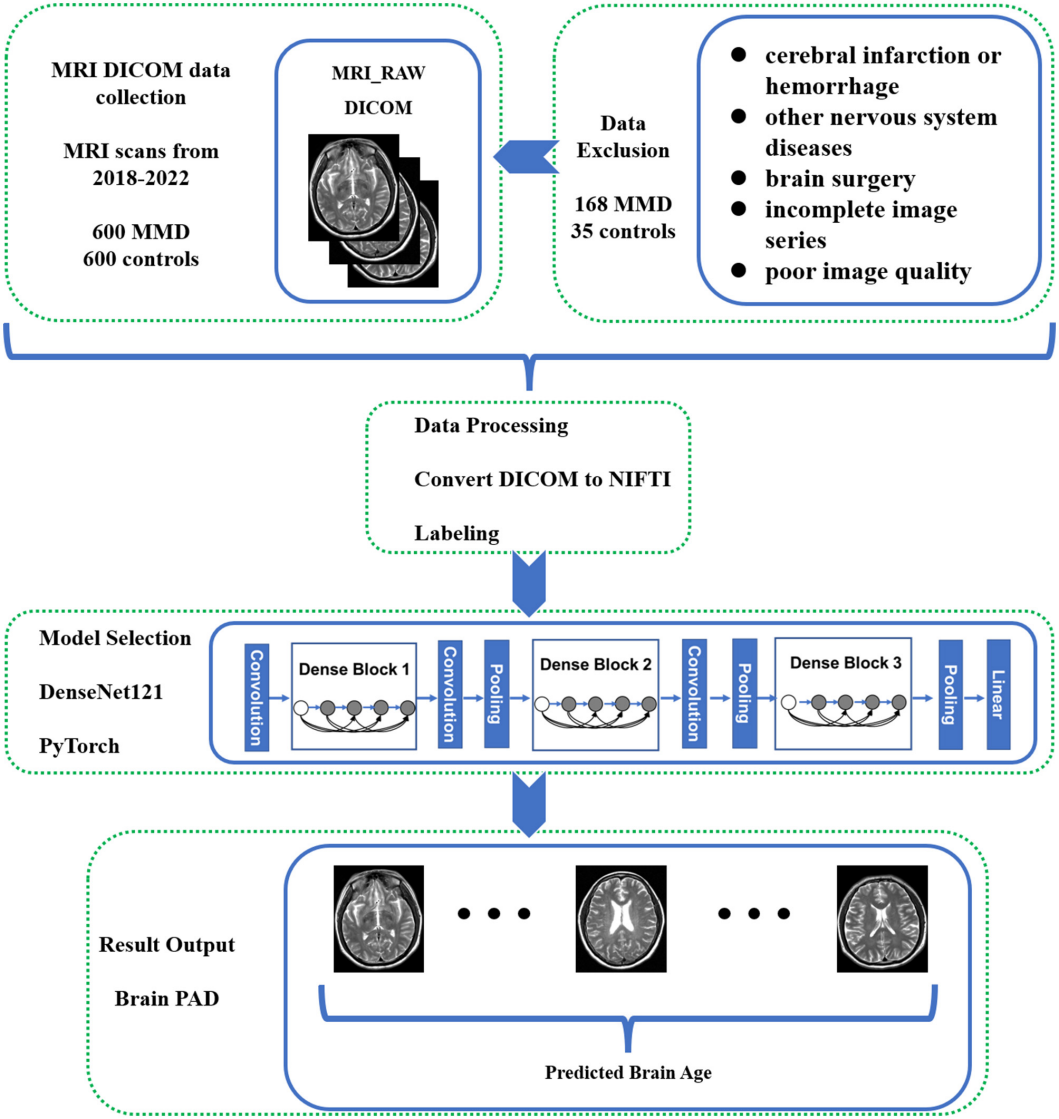
Flowchart of the study methodology. This flowchart illustrates the step-by-step methodology employed in the study for predicting brain age in Moyamoya disease (MMD) patients using deep learning techniques.

The unenhanced MRI scans of 600 adult MMD patients and normal individuals at hospital between January 2018 and December 2024 (Figure 1). The MRI scans were performed on Signa 3.0T (General Electric Healthcare, Chicago, US), Ingenia 3.0 T (Philips Healthcare, Eindhoven, Netherlands) or Verio 3 T (Siemens, Erlangen, Germany) scanners.

We excluded patients who had been diagnosed (1) with cerebral infarction or cerebral hemorrhage, (2) had other cerebrovascular or nervous system diseases, (3) had a history of brain surgery, (4) had incomplete image series, or (5) had poor image quality due to artifacts or a low signal-to-noise ratio. Of the initial 600 patients, only 432 met the criteria for inclusion in the model development dataset. For the control group, we initially reviewed 600 candidate cases who underwent MRI for routine health examinations or non-cerebrovascular complaints. Individuals with any history of cerebrovascular or neurological disease were excluded. After removing 35 cases with incomplete sequences or poor image quality, 565 normal controls were retained.

This study was approved by the Institutional Review Board (IRB) of hospital, and all medical images and clinical data were fully anonymized. Digital Imaging and Communications in Medicine (DICOM) images were obtained from Picture Archiving and Communication System (PACS) servers in compliance with the Health Insurance Portability and Accountability Act.

### Data labeling

The age of all patients was extracted from the header information of MRI scans using PyDicom, a Python package for working with DICOM files. Batch processing techniques were employed to ensure efficient extraction and compilation of patient demographic data.

The diagnostic criteria for definite MMD were in accordance with the guidelines by the Research Committee on MMD of Japan (Kuroda et al., 2022). The diagnosis of MMD was confirmed using additional clinical evidence, including CTA, MRA, and DSA. The magnetic resonance angiography (MRA) images of patients with Moyamoya disease were subjected to detailed analysis. The severity of arterial stenosis or occlusion was evaluated using the Houkin MRA scoring system. In this system, the Houkin MRA scoring system for Moyamoya disease assigns a score to each major cerebral artery based on its stenosis or signal status on MRA. For the internal carotid artery (ICA), a normal vessel is scored 0, C1 segment narrowing 1, C1 segment signal interruption 2, and disappearance 3. For the middle cerebral artery (MCA), a normal vessel is 0, M1 segment narrowing is 1, M1 segment signal interruption is 2, and disappearance is 3. For the anterior cerebral artery (ACA), the A2 segment and its distal portion are scored 0 if normal, 1 if the signal is reduced, and 2 if the segment is absent. For the posterior cerebral artery (PCA), the P2 segment and its distal portion are scored 0 if normal, 1 if the signal is reduced, and 2 if absent. The total score ranges from 0 to 10, with higher scores indicating more severe vascular involvement. Specifically, MRA images were evaluated for the severity and distribution of stenosis or occlusion within the cerebral arteries using established scoring system. To ensure accurate classification, two experienced neuroradiologists (each with >10 years of experience) independently reviewed the MRI/MRA scans to confirm the absence of Moyamoya disease or other brain abnormalities. Any discrepancies were resolved by consensus with a third senior radiologist.

### Data preprocessing

The raw data collected is in DICOM format, and it has been organized into different folders based on their respective labels. The T2WI series data has been converted into neuroimaging informatics technology initiative (NIFTI) format and renamed using dcm2niigui software (v2.1.64-1, MRIcron, McCausland Center for Brain Imaging, United States).

### Model selection

In this study, we employed a brain-age prediction model based on the DenseNet121 architecture, as described by David A. Wood et al. The DenseNet architecture, which incorporates shortcut connections between internal neuron layers, was chosen to mitigate the vanishing gradient problem commonly encountered in deep neural networks (Pascanu et al., 2013). The DenseNet brain-age models were adapted from the implementation available at Project MONAI, utilizing PyTorch 1.7.1 (Paszke et al., 2019). Scripts enabling replication of brain-age prediction models using custom scans are available at the provided GitHub repository (https://github.com/MIDIconsortium/BrainAge). All modeling was performed with an NVIDIA RTX 4090 24 GB graphics processing units (GPU).

### Statistical analysis

The categorical variables are represented by counts while the continuous variables are represented by means ± standard deviations. Categorical variables were compared using Fisher's exact test, Pearson's chi-square test, or the Mann-Whitney U-test. Differences in the mean of continuous variables between groups were analyzed using the student's t-test. The results were considered statistically significant when the probability value was < 0.05. All statistical analyses were performed using SPSS software (Version 26.0, IBM, New York, NY, USA).

## Results

The study analyzed data from 432 Moyamoya disease (MMD) patients and 565 normal controls, after excluding cases with incomplete sequences or poor image quality.

### Demographic and imaging data

As shown in Table 1, the average age of MMD patients was 35.8 years, with a gender distribution of 195 males and 237 females. MRI data for these patients were sourced from SIEMENS (196), General Electric Healthcare (169), and Philips Healthcare (67). For the normal controls, the average age was 36.5 years, with 252 males and 313 females. Their MRI data came from SIEMENS (234), General Electric Healthcare (187), and Philips Healthcare (144).

**Table 1:** Demographic and imaging characteristics of participants with MMD and controls.

**Participant**	**MMD *n***	**Controls *n***
Number	432	565
Age (mean ± standard deviation)	35.84 ± 18.97	36.53 ± 8.08
Male/female	195/237	252/313
Manufacturer		
SIEMENS	196	234
General electric healthcare	169	187
Philips healthcare	67	144

MMD, Moyamoya disease; n, number of patients.

### Predicted brain age analysis

**Figure 2:**
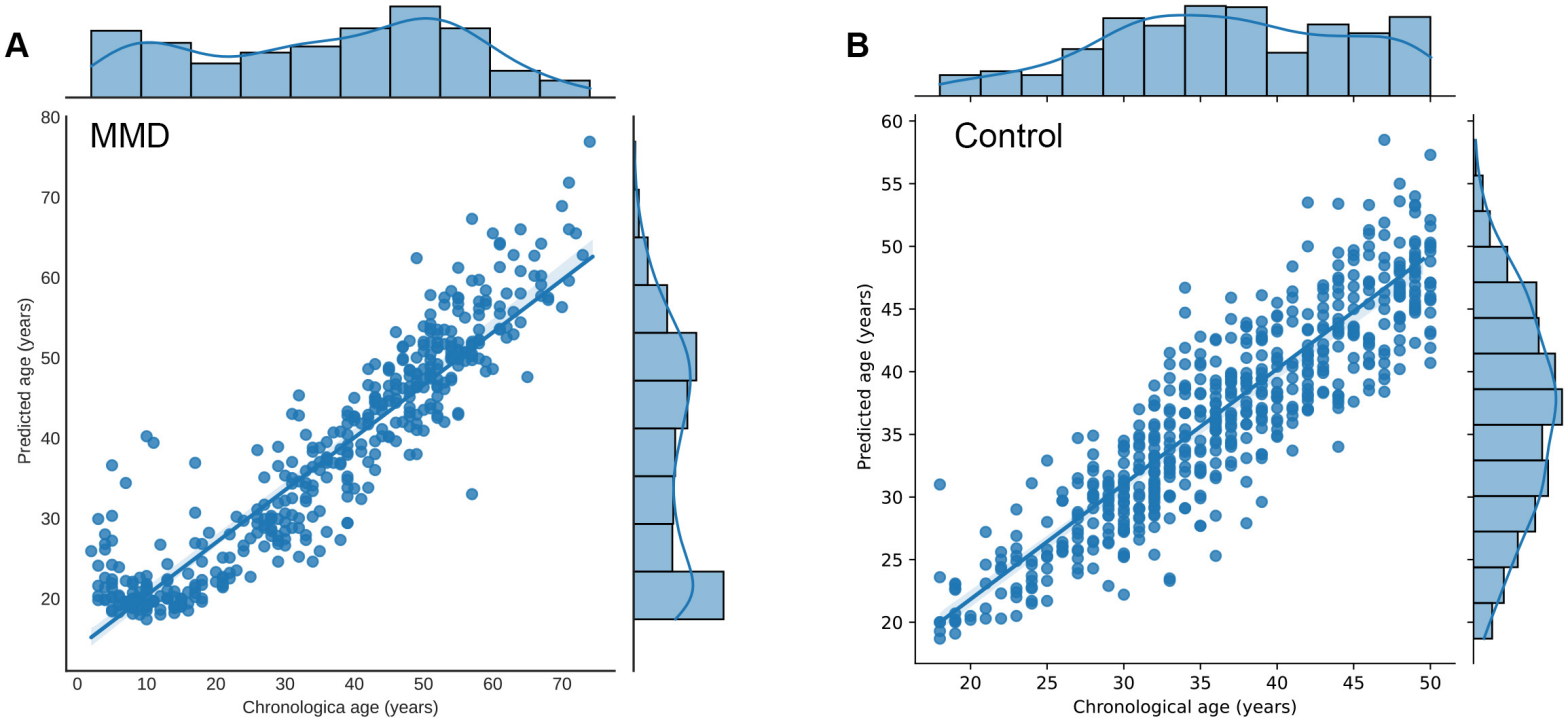
Relationship between predicted brain age and chronological age in participants. **(A)** Correlation analysis revealed a significant positive relationship between predicted brain age and chronological age in MMD patients (*r* = 0.77, *p* < 0.01). **(B)** Correlation analysis revealed a significant positive relationship between predicted brain age and chronological age in controls (*r* = 0.89, *p* < 0.01). Each point represents the age of participants, with the x-axis showing the chronological age (years) and the y-axis showing the corresponding predicted age (years). The marginal histograms along the top and right edges of the plot display the chronological age and predicted brain age, respectively. The plot also includes a linear regression line (not shown in the example code) with a 95% confidence interval, further emphasizing the positive correlation.

As shown in Table 2, The average predicted brain age for MMD patients was 37.9 years, compared to their average chronological age of 35.8 years. Correlation analysis revealed a significant positive relationship between predicted brain age and chronological age in MMD patients (*r* = 0.77, *p* < 0.01, Figure 2A), indicating that the model effectively captures brain aging in this group.

**Table 2:** Predicted vs. chronological age and correlation in MMD patients and controls.

**Participant**	**Predicted age (mean ±standard deviation)**	**Chronological age (mean ±standard deviation)**	**Correlation coefficient**	***p*-value**
MMD	37.92 ± 13.72	35.84 ± 18.97	0.773^**^	0.001
Controls	36.52 ± 8.10	36.53 ± 8.08	0.894^**^	

MMD, Moyamoya disease; ^*^p < 0.05; ^**^p < 0.001.

For normal controls, the average predicted brain age matched their chronological age at 36.5 years, with a stronger correlation (*r* = 0.89, *p* < 0.01, Figure 2B), confirming the model's accuracy in estimating brain age for healthy individuals.

### Delta age analysis

**Figure 3:**
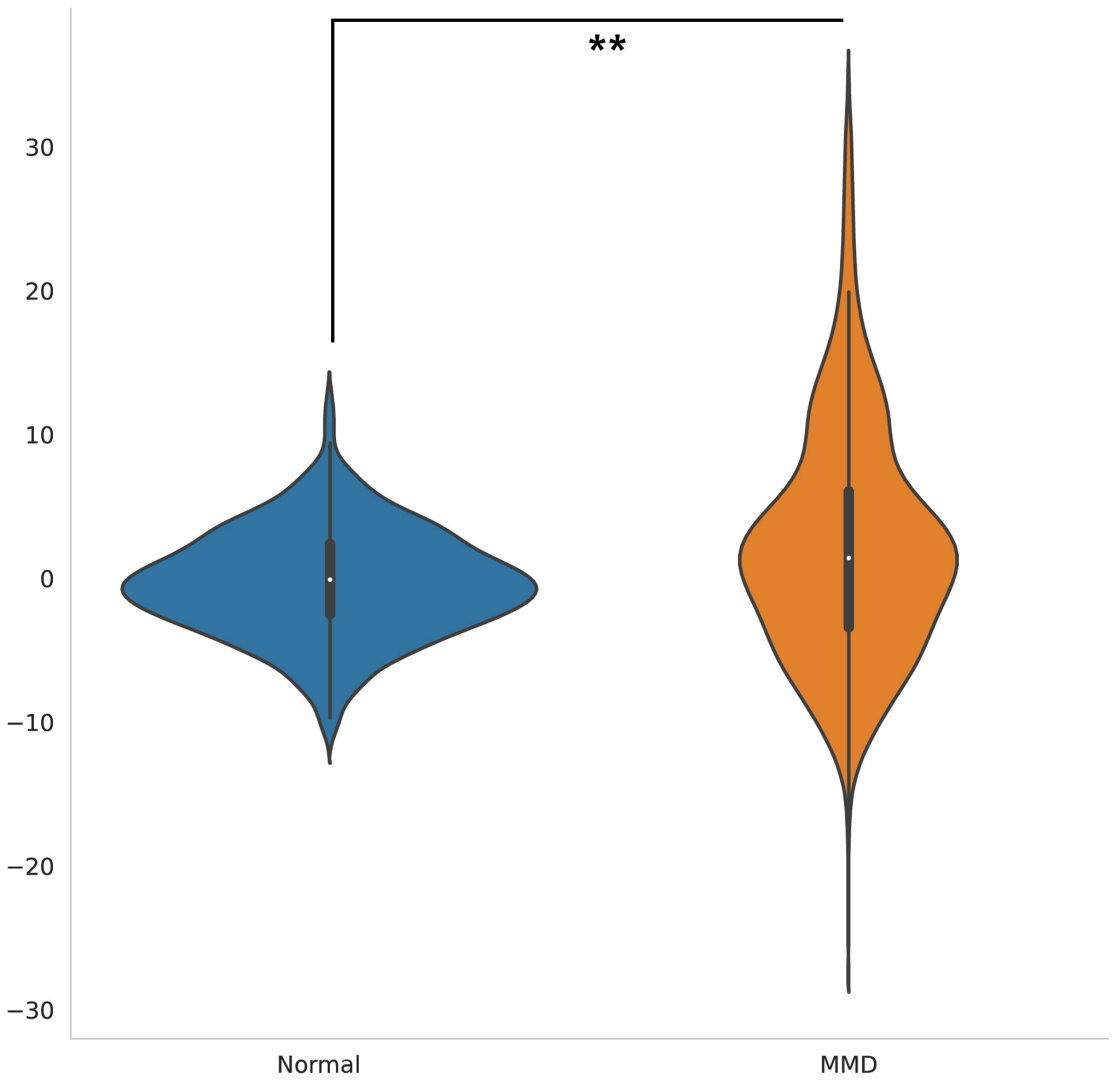
Violin plots illustrate the distribution of delta age for MMD patients and controls. The delta age was significantly higher in MMD patients than in normal controls (*p* < 0.001). The white dot signifies the median, while the thick horizontal line denotes the interquartile range (IQR).

Delta age, defined as the difference between predicted brain age and chronological age, was significantly higher in MMD patients compared to normal controls (*p* < 0.001, Figure 3). This suggests accelerated brain aging in individuals with Moyamoya disease.

Further analysis demonstrated a significant positive correlation between delta age and the highest MRA score (*p* < 0.01) as well as the average MRA score (*p* < 0.05) in MMD patients (Table 3). These findings indicate that greater arterial stenosis or occlusion severity is associated with more pronounced brain aging.

**Table 3:** Association between MRA scores and delta age in MMD patients.

**MRA score**	**Rank**	**Δ age (mean ±standard deviation)**	**Correlation coefficient**
Average score	6.07	2.07 ± 8.01	0.141^**^
Max score	6.85	2.07 ± 8.01	0.117^*^

MRA, magnetic resonance angiography; Δage, delta age; ^*^p < 0.05; ^**^p < 0.001.

## Discussion

The study's findings reveal significant insights into the relationship between MMD and brain aging. The application of a DenseNet-based deep learning model to predict brain age demonstrated that MMD patients experience accelerated brain aging compared to normal controls. The positive correlation between delta age and MRA scores suggests that the severity of arterial stenosis or occlusion is closely linked with increased brain aging. These results highlight the potential utility of brain age prediction as a biomarker for assessing disease severity and progression in MMD patients.

Accurate brain age estimation can assist clinicians in classifying MMD patients based on the severity of age-related symptoms, aiding in the development of personalized treatment strategies. Early detection through brain age prediction may promote timely intervention, potentially preventing serious complications such as stroke. This study underscores the importance of integrating advanced neuroimaging techniques and machine learning models in clinical practice to enhance diagnostic and prognostic capabilities.

Previous research has established the impact of cerebral hypoperfusion on cognitive function and brain structure in MMD patients (Su et al., 2013; Kazumata et al., 2019; Festa et al., 2010). Studies have shown that decreased gray matter volume is associated with cognitive decline in these individuals (Zimmerman et al., 2006). Our findings align with this body of literature by demonstrating that MMD patients exhibit higher predicted brain ages compared to their chronological ages, reflecting accelerated brain aging.

The utilization of deep learning models, particularly DenseNet, for brain age prediction represents a significant advancement over traditional methods. Previous studies have employed machine learning techniques to estimate brain age; however, these models often require high-resolution images and extensive data preprocessing (Baecker et al., 2021). Our approach using DenseNet efficiently handles large, high-dimensional datasets, providing accurate brain age predictions with routine clinical MRI examinations. This enhances the feasibility of incorporating brain age prediction into standard clinical workflows.

Despite the promising results, several limitations should be acknowledged. First, the study's retrospective design and the use of data from a single institution may limit the generalizability of the findings. The sample size, while substantial, may not capture the full spectrum of variability in MMD patients and normal controls.

Second, the exclusion of patients with incomplete image series or poor image quality might introduce selection bias, potentially affecting the model's performance in real-world clinical settings.

Future research should focus on addressing the limitations identified in this study. Expanding the dataset to include multi-center data and diverse patient populations will enhance the generalizability and robustness of the findings. Incorporating longitudinal data will allow for the investigation of causal relationships between brain aging and disease progression in MMD patients.

Additionally, future studies should explore the integration of multimodal imaging data, such as combining T1-weighted, T2-weighted, and functional MRI, to provide a more comprehensive assessment of brain structure and function. This could further improve the accuracy and reliability of brain age prediction models.

Moreover, investigating the potential therapeutic implications of brain age prediction in MMD patients is crucial. In the future, brain age prediction may help choose the timing of interventions. Understanding how interventions such as cerebral revascularization impact brain aging could inform treatment strategies and improve patient outcomes.

## Conclusions

In conclusion, this study demonstrates the utility of a DenseNet based deep learning model for predicting brain age in MMD patients, revealing significant associations between brain aging and disease severity. These findings have important implications for the diagnosis, prognosis, and treatment of Moyamoya disease, paving the way for future research to build upon and refine these initial insights.

## Data Availability

The raw data supporting the conclusions of this article will be made available by the authors, without undue reservation.
